# Medical Miscellany

**Published:** 1846-07

**Authors:** 


### MEDICAL MISCELLANY.

Dr. McDowell has withdrawn from the Missouri Medical Journal, and Dr. Barbour is now its sole editor. The Illinois Medical Journal, much enlarged, and improved in appearance, is to be issued from this time forward bi-monthly. Dr. J. W. Hall has resigned the Chair of the Institutes of Medicine in the St. Louis University, and is succeeded by Dr. Henry M. Bullitt, of Louisville. Robert Chambers, the great bookseller and publisher of Edinburg, has avowed himself the author of "Vestiges of Creation." The authorship has generally been ascribed to Sir Richard Vyvyan; but in an elaborate article in the American Review an attempt was made, some months ago, to prove, by similarity of style, that it was the production of Taylor, the author of Saturday Evening, the Natural History of Enthusiasm, &c. A *second* medical school is spoken of in Memphis, as a department of the Memphis University.

